# Plant-produced chimeric virus-like particles - a new generation vaccine against African horse sickness

**DOI:** 10.1186/s12917-019-2184-2

**Published:** 2019-12-03

**Authors:** Daria A. Rutkowska, Nobalanda B. Mokoena, Tsepo L. Tsekoa, Vusi S. Dibakwane, Martha M. O’Kennedy

**Affiliations:** 1 CSIR Chemicals, PO Box 395, Pretoria, 0001 South Africa; 2 Onderstepoort Biological Products SOC Ltd, Private Bag X07, Onderstepoort, 0110 South Africa

**Keywords:** Plant-expressed, Chimeric, Virus-like particle, VLP, Vaccine, African horse sickness, AHSV, *Orbivirus*

## Abstract

**Background:**

African horse sickness (AHS) is a severe arthropod-borne viral disease of equids, with a mortality rate of up to 95% in susceptible naïve horses. Due to safety concerns with the current live, attenuated AHS vaccine, alternate safe and effective vaccination strategies such as virus-like particles (VLPs) are being investigated. Transient plant-based expression systems are a rapid and highly scalable means of producing such African horse sickness virus (AHSV) VLPs for vaccine purposes.

**Results:**

In this study, we demonstrated that transient co-expression of the four AHSV capsid proteins in agroinfiltrated *Nicotiana benthamiana* dXT/FT plants not only allowed for the assembly of homogenous AHSV-1 VLPs but also single, double and triple chimeric VLPs, where one capsid protein originated from one AHS serotype and at least one other capsid protein originated from another AHS serotype. Following optimisation of a large scale VLP purification procedure, the safety and immunogenicity of the plant-produced, triple chimeric AHSV-6 VLPs was confirmed in horses, the target species.

**Conclusions:**

We have successfully shown assembly of single and double chimeric AHSV-7 VLPs, as well as triple chimeric AHSV-6 VLPs, in *Nicotiana benthamiana* dXT/FT plants. Plant produced chimeric AHSV-6 VLPs were found to be safe for administration into 6 month old foals as well as capable of eliciting a weak neutralizing humoral immune response in these target animals against homologous AHSV virus.

## Background

African horse sickness (AHS) is a devastating disease of equids causing severe morbidity in naïve horses with the mortality rate up to 95% [1]. Although donkeys, mules and zebras may also be infected, clinical signs in susceptible horses range from mild to high fever, severe respiratory problems, weight loss and lethargy in the acute form [2]. Although AHS is endemic to sub-Saharan Africa, outbreaks have also been reported in North Africa, the Iberian Peninsula, the Middle East and Asia and, due to climate change, there is an ever-increasing risk of the spread of the disease, and its vectors, to non-endemic countries [3]. The disease is included in the World Organisation for Animal Health (OIE) 2019 list of notifiable animal diseases, resulting in strict quarantine measures governing the export of horses from endemic to non-endemic regions. This places a significant economic burden on the equine industries of affected countries.

African horse sickness virus (AHSV), the causative agent of AHS, is an *Orbivirus* in the family *Reoviridae* and is transmitted to susceptible animals via *Culicoides* midges [4]. The AHS virion is a 70 nm icosahedral, non-enveloped particle, composed of three concentric layers surrounding the segmented double-stranded RNA genome [1, 5]. Like *Bluetongue virus* (BTV), the prototype *Orbivirus* [6], the subcore of AHSV is composed of 120 copies of structural protein VP3 and is covered by a layer of VP7 trimers forming the core particle. The core is surrounded by the outermost layer composed of structural proteins VP5 and VP2, with VP2 being the neutralizing antigen and serotype determinant [7–10]. There are nine AHSV serotypes present within South Africa and most parts of sub-Saharan Africa [1]. In these endemic regions, annual prophylactic vaccination of horses with a commercial live attenuated vaccine (Onderstepoort Biological Products (OBP)) is an efficient way of preventing serious losses during the peak AHS season [11]. There are, however, several drawbacks associated with the use of the current live AHS vaccine including the risk of reversion to virulence, genome segment reassortment between vaccine and field strains and teratogenic effects in pregnant mares [2, 12, 13]. There is also no way of differentiating between infected and vaccinated animals (DIVA). Thus there is an urgent need for safe, efficacious and cost-effective new generation vaccines against AHS for use in endemic as well as non-endemic countries. New AHS vaccine candidates, based on subunit vaccine, recombinant viral vectored vaccine and reverse genetics approaches have been developed but each have their own disadvantages and have not yet progressed to the market [9, 14–24].

Virus-like particles (VLPs) are considered a safe and effective alternative to live attenuated vaccines for many viral diseases [25]. These self-assembling particles, composed of viral structural proteins but lacking the viral genome, exhibit size and morphology very similar to that of native virions but are unable to replicate [25–28]. Such VLP-based vaccines are inherently safe because they are unable to replicate or reassort as they do not contain a genome and are DIVA-compliant due to their lack of non-structural proteins. Co-expression of BTV VP2, VP5, VP7 and VP3 capsid proteins in insect cells resulted in the assembly of VLPs capable of eliciting a long-lasting protective immune response against virulent BTV challenge in vaccinated sheep [29, 30]. Recently co-expression of the four AHSV-9 capsid proteins in insect cells allowed for self-assembly of AHS VLPs [31]. However, the yield of these assembled AHS VLPs was very low, precluding their quantification. Due to the low yield and high costs associated with large scale production of *Orbivirus* VLPs in the insect cell expression system, it is unlikely that this expression system will be utilised to produce *Orbivirus* VLP-based vaccines, other than monovalent candidates, on a commercial scale.

Transient expression in plants is currently being employed for the fast and relatively easy production of VLPs, antibodies and other heteromultimeric complexes [32, 33]. Advantages of transient plant expression systems include speed, scalability, cost-effectiveness and safety [34–39]. Transient co-expression of the structural proteins of Bluetongue virus in plant cells [40, 41] allowed for the self-assembly of BTV-8 VLPs that elicited a protective immune response against virulent BTV-8 challenge in sheep [42, 43]. Recently, the four capsid proteins VP2, VP3, VP5 and VP7 of AHSV-5 were shown to self-assemble into homogenous VLPs when transiently expressed in *Nicotiana benthamiana* plants [44]. These plant-produced AHSV-5 VLPs were also shown to elicit strong neutralizing immune responses in both guinea pigs and horses [45]. An RNAi mutant dXT/FT *N. benthamiana*, which facilitates mammalian-like glycosylation [46] and may result in AHSV VLPs more closely resembling AHSV virions with authentic post translational modifications, is available in our laboratory.

As *Orbivirus*es are capable of genome segment reassortment, one of the strategies of generating serotype-specific VLP-based vaccine candidates is to exchange the outer capsid proteins VP2 and/or VP5 with those of a different serotype whilst retaining the conserved inner core (VP7 and VP3 proteins) of the original serotype [47, 48]. This strategy of generating such ‘chimeric’ VLPs would reduce the number of constructs required and significantly simplify the process of generating a multivalent vaccine. Replication-deficient chimeric BTV and AHSV have recently been generated via a reverse genetics approach by exchanging specific gene segments with those of another serotype [49–52].

In this study, we report on the transient expression and self-assembly of both homogenous, as well as single, double and triple chimeric AHS VLPs in *N. benthamiana* dXT/FT plants. We also demonstrate that plant-produced, chimeric AHSV-6 VLPs are safe and immunogenic in horses, the target species, and capable of eliciting neutralizing antibodies against homologous AHSV-6 virus.

## Results

### Transient capsid protein expression and assembly of chimeric AHS VLPs in plants

In order to facilitate the assembly of African horse sickness virus-like particles (VLPs) in *N. benthamiana* dXT/FT plant cells, sequences encoding the AHS structural capsid proteins VP2, VP5, VP3 and VP7 were codon-optimised for *N. benthamiana* expression and cloned into either the pEAQ-HT or pEAQ-express plant expression vectors [40]. The AHSV-1 VP7 and VP3-encoding genes were inserted into the same pEAQ-express vector to ensure co-expression and also reduce the number of recombinant plasmids required for co-infiltration.

**Fig. 1:**
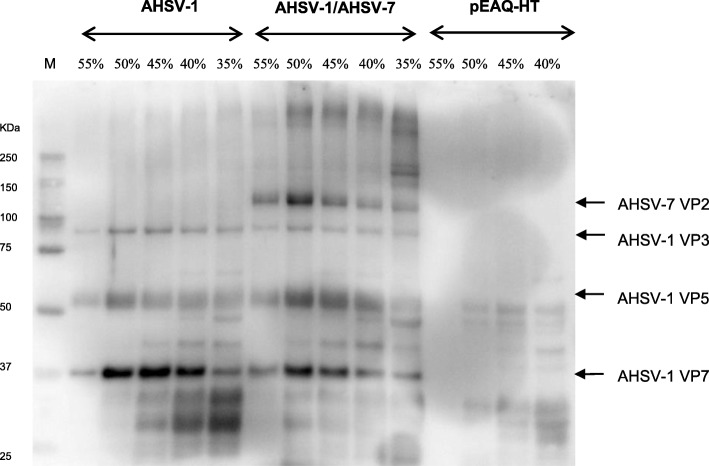
Immunoblot detection of AHSV-1 and/or AHSV-7 capsid proteins in 55–35% sucrose fractions. Precision Plus Protein™ Western C™ standard (Bio-Rad) is the marker (M) and the relevant sizes are indicated. Fractions of the AHSV-1 gradient are present in lanes 2–6, while fractions of the AHSV-1/AHSV-7 gradient and the pEAQ-HT gradient (negative control) are present in lanes 7–11 and lanes 12–15, respectively. Indicated above the lanes are the sucrose concentrations of the fractions. Immunoblotting was performed with a guinea pig anti-AHSV-7 serum. Arrows indicate the position of the AHSV-7 VP2 (123,6 KDa), AHSV-1 VP3 (103,2KDa), AHSV-1 VP5 (56.6KDa) and AHSV-1 VP7 (37.8KDa) proteins on the immunoblot membrane

**Fig. 2:**
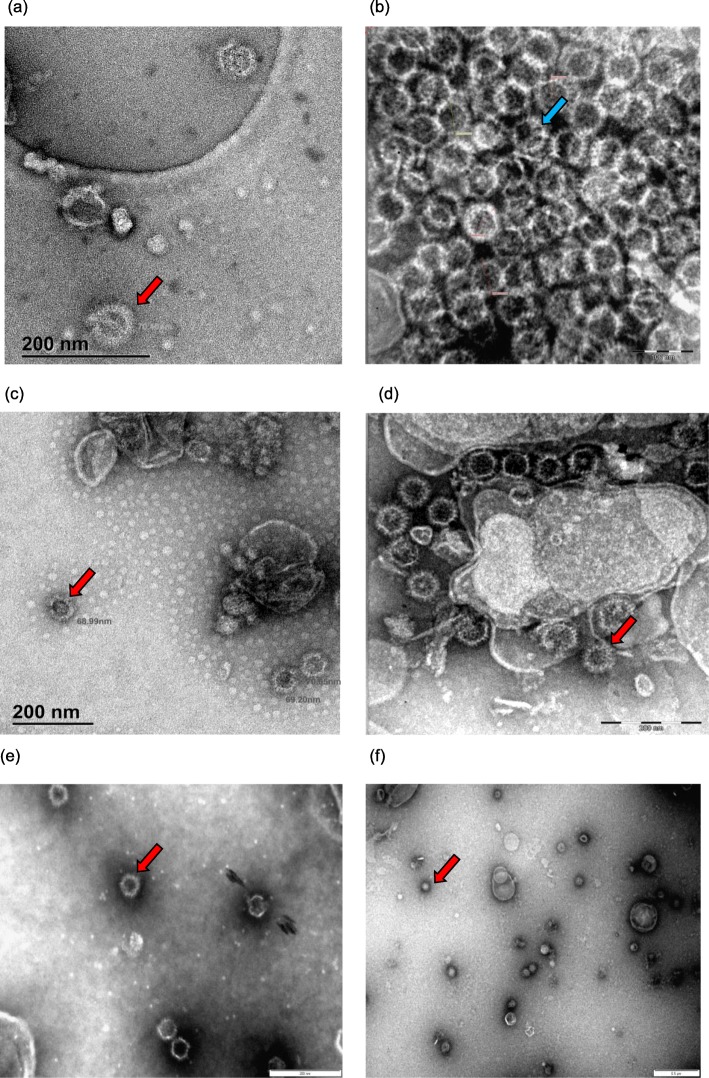
Transmission electron microscope (TEM) images of density gradient purified AHSV-1 VLPs (**a**), AHSV-1 CLPs (**b**), chimeric AHSV-1/AHSV-7 VLPs (**c**), double chimeric AHSV-1/AHSV-7 VLPs (**d**) and triple chimeric AHSV-1/AHSV-6/AHSV-3 VLPs (**e**-**f**). Particles were visualized with a JEM-2100 Transmission electron microscope (JEOL). Indicated with red arrows are the virus-like particles (VLPs) and indicated with the blue arrow is a core-like particle (CLP)

*N. benthamiana* dXT/FT leaves were infiltrated with different combinations of the recombinant *Agrobacterium tumefaciens* bacteria. In order to facilitate the assembly of the homogenous AHSV-1 VLPs, the pEAQ-HT-AHSV-1VP2, pEAQ-express-AHSV-1VP5 and pEAQ-express-AHSV-1VP7-AHSV-1VP3 plasmids were agroinfiltrated into *N. benthamiana* dXT/FT leaves in a ratio of 1:1:1. Assembly of AHS core-like particles (CLPs) was facilitated by the agroinfiltration of the dual plasmid pEAQ-express-AHSV-1VP7-AHSV-1VP3. Infiltrated leaves were harvested 8 days post-infiltration, as previously reported for optimal BTV capsid protein transient expression [42, 43], and the leaf tissue extract centrifuged through sucrose density gradients. The presence of the AHSV-1 capsid proteins within the sucrose fractions was assessed by SDS-PAGE and immunoblotting with AHS serotype 7-specific antiserum (Fig. 1). The AHSV-7 antibodies bound to protein bands corresponding in size to the AHSV-1 VP3 (103.2KDa), VP5 (56.6KDa) and VP7 (37.8KDa) capsid proteins in the 55–35% sucrose fractions (Fig. 1, lanes 2–6). These bands were not visible in lanes 12–15, containing 55–40% sucrose fractions from the pEAQ-HT cell lysate gradient, the negative control. The identities of the AHSV-1 VP5, VP7 and VP3 proteins were confirmed using LC-MS/MS-based peptide sequencing. The presence of all three capsid proteins within the same sucrose fractions following centrifugation indicates that these proteins are assembling into high molecular weight particulate structures. Although not detected by the AHSV-7 serotype-specific antiserum, the AHSV-1 VP2 neutralizing antigen (123.6 KDa) is thought to be part of these particulate structures. To view these particulate structures, a sample of the 55% sucrose gradient fractions was stained with uranyl acetate and viewed under the transmission electron microscope (TEM) (Fig. 2a). The AHSV-1 particulate structures resembled virus-like particles (VLPs) previously described [31]. These AHSV-1 VLPs were approximately 70 nm in diameter and appeared more densely populated than the ‘spiky’, knob-like surface of the 60 nm AHSV-1 core like particles (CLPs), consisting only of the VP7 and VP3 core proteins (Fig. 2b).

As AHS has nine serologically distinct serotypes, a multivalent vaccine is required in order to protect against this disease. Thus it was decided to exchange the outer capsid AHSV-1 VP2 protein with that of the AHSV-7 serotype whilst retaining the AHSV-1 VP5 and conserved AHSV-1 VP7 and VP3 core proteins. This strategy would reduce the number of constructs required and significantly simplify the process of generating a multivalent VLP-based AHS vaccine in plants. The pEAQ-HT-AHSV-7VP2, pEAQ-express-AHSV-1VP5 and pEAQ-express-AHSV-1VP7-AHSV-1VP3 plasmids were agroinfiltrated into *N. benthamiana* dXT/FT leaves in a ratio of 1:1:1. Infiltrated leaves were harvested 8 days post-infiltration, as previously, and the leaf tissue extract centrifuged through sucrose density gradients. The presence of the AHSV-1/AHSV-7 capsid proteins within the sucrose fractions was assessed by SDS-PAGE and immunoblotting with AHS serotype 7-specific antiserum (Fig. 1). Not only did the AHSV-7 antibodies bind to protein bands corresponding in size to the AHSV-1 VP3 (103,2KDa), VP5 (56.6KDa) and VP7 (37.8KDa) capsid proteins in the 55–35% sucrose fractions, but also to the band corresponding in size to the AHSV-7 VP2 neutralizing antigen (123.6 KDa) (Fig. 1, lanes 7–11). These bands were not visible in lanes 12–15, containing 55–40% sucrose fractions from the pEAQ-HT cell lysate gradient, the negative control. The presence of all four capsid proteins in these sucrose fractions indicate their assembly into high molecular weight particulate structures. TEM visualisation of the particles in sucrose fraction 5 revealed the presence of densely structured single chimeric AHSV-1/AHSV-7 VLPs, approximately 70 nm in size (Fig. 2c).

**Fig. 3:**
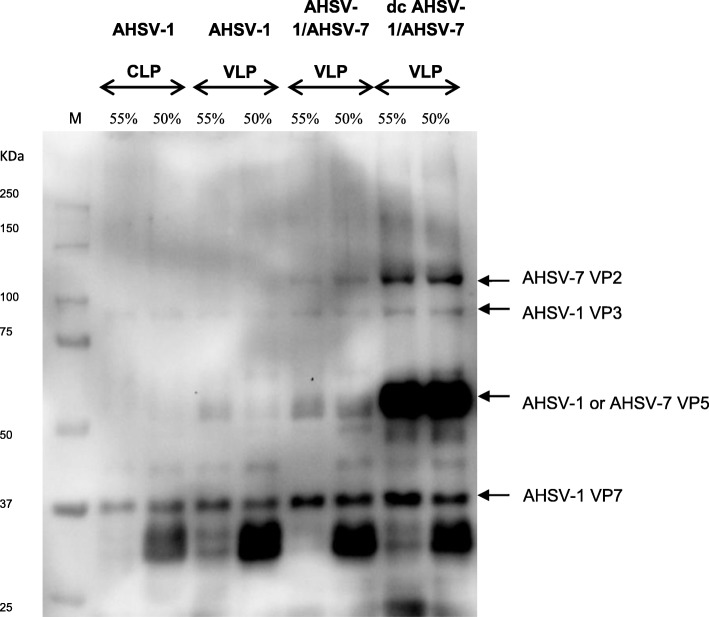
Immunoblot detection of AHSV-1 and/or AHSV-7 capsid proteins in 55–50% sucrose fractions. Precision Plus Protein™ Western C™ standard (Bio-Rad) is the marker (M) and the relevant sizes are indicated. Fractions of the AHSV-1 CLP gradient, the AHSV-1 VLP gradient, chimeric AHSV-1/AHSV-7 VLP gradient and double chimeric (dc) AHSV-1/AHSV-7 VLP gradient are present in lanes 2–3, 4–5, 6–7 and 8–9, respectively. Indicated above the lanes are the sucrose concentrations of the fractions. Immunoblotting was performed with a guinea pig anti-AHSV-7 serum. Arrows indicate the position of the AHSV-7 VP2 (123,6KDa), AHSV-1 VP3 (103.2KDa), AHSV-1 VP5 or AHSV-7 VP5 (56.6KDa) and AHSV-1 VP7 (37.8KDa) proteins on the immunoblot membrane

Next, it was investigated whether both AHSV-1 outer capsid proteins VP2 and VP5 could be exchanged with those of AHSV-7 whilst allowing for the assembly of double chimeric AHS VLPs in plants. Genes encoding the AHSV-7 VP2 and VP5 proteins were co-infiltrated into *N. benthamiana* dXT/FT plant cells with those encoding the AHSV-1 VP3 and VP7 proteins. Single chimeric AHSV-1/AHSV-7 VLPs were expressed as a positive control. Following harvesting of the leaves 8 days post-infiltration, extraction and density gradient centrifugation, the presence of the AHSV capsid proteins within the sucrose fractions was confirmed by SDS-PAGE and immunoblotting with AHS serotype 7-specific antiserum (Fig. 3). Visualisation with the TEM confirmed the presence of double chimeric AHSV-1/AHSV-7 VLPs, 70 nm in size (Fig. 2d).

**Fig. 4:**
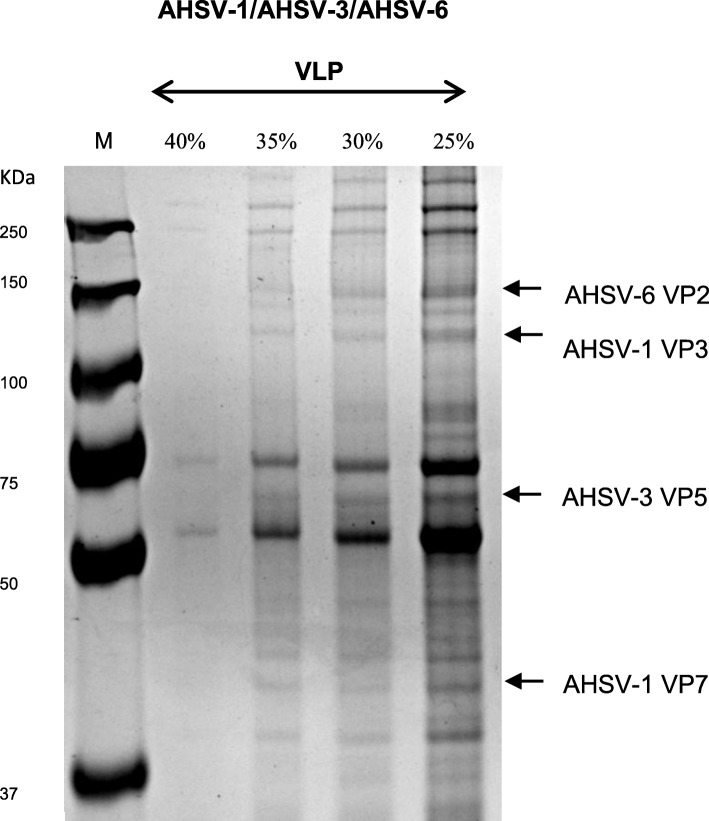
SDS-PAGE of the AHSV-1, AHSV-3 and AHSV-6 capsid proteins in 40–25% iodixanol fractions. The PageRuler™ Plus Prestained protein ladder (Thermo Scientific) is the marker (M) and the relevant sizes are indicated. The 40, 35, 30 and 25% fractions of the triple chimeric AHSV-1/AHSV-3/AHSV-6 VLP gradient are present in lanes 2–5, respectively. Indicated above the lanes are the iodixanol concentrations of the fractions. Arrows indicate the position of the AHSV-6 VP2 (123,6KDa), AHSV-3 VP5 (56.6KDa), AHSV-1 VP3 (103.2KDa) and AHSV-1 VP7 (37.8KDa) proteins on the gel

**Fig. 5:**
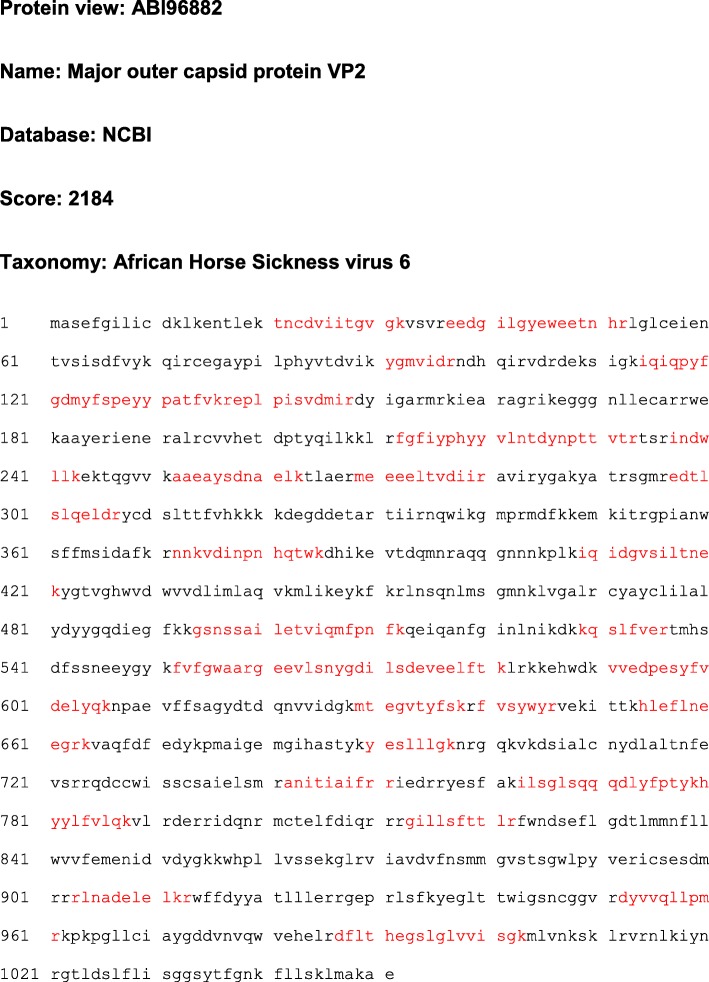
LC-MS/MS based peptide sequencing analysis of putative VP2 protein bands excised from an SDS-PAGE gel. *N.benthamiana* leaves, co-infiltrated with constructs encoding the AHSV-6 VP2, AHSV-3 VP5, AHSV-1 VP3 and AHSV-1 VP7 proteins, were harvested and the extracted cell lysate centrifuged through a density gradient. Selected gradient fractions were electrophorized on an SDS-PAGE gel and the putative gel bands excised for MS analysis. Matched peptides are shown in red. Scores > 75 are considered significant (*p* < 0.05)

We proceeded to investigate combining the VP2 and VP5 proteins of different serotypes with the AHSV-1 core proteins. In one of these experiments, the pEAQ-HT-AHSV-6VP2, pEAQ-HT-AHSV-3VP5 and pEAQ-express-AHSV-1VP7-AHSV-1VP3 plasmids were agroinfiltrated into *N. benthamiana* dXT/FT leaves in a ratio of 1:1:1. Eight days post inoculation, infiltrated leaves were extracted in VLP dilution buffer and density gradient centrifugation, and the presence of the AHSV capsid proteins within the gradient fractions assessed by SDS-PAGE (Fig. 4). Protein bands corresponding in size to the AHSV-6 VP2 (123.6KDa), AHSV-3 VP5 (56.6KDa), AHSV-1 VP3 (103.2KDa) and AHSV-1 VP7 (37.8KDa) capsid proteins were observed in the 35–25% iodixanol fractions (Fig. 4, lanes 3–5). The identity of AHSV-6 VP2 protein band was confirmed by LC-MS/MS-based peptide sequencing (Fig. 5). LC-MS/MS analysis of VP2 bands in lanes 4 and 5 (Fig. 4) resulted in 63.7% coverage for VP2, with 84 peptides identified with > 95% confidence, and 55.8% coverage for VP2, with 85 peptides identified with > 95% confidence in the VLPs produced, respectively.

The presence of all four AHS capsid proteins within the same density gradient fractions was indicative of their assembly into high molecular weight particles. Particles in the 30% iodixanol fraction were viewed via TEM (Fig. 2e-f). Triple chimeric VLPs, approximately 70 nm in size, were visualised.

### Safety and immunogenicity of plant-produced chimeric AHS VLPs in animals

Plant-produced triple chimeric AHSV-1/AHSV-3/AHSV-6 VLPs were purified using a combination of depth filtration and tangential flow filtration (TFF). The yield after filter sterilization was approximately 16 mg VLPs per kg wet leaf weight as determined by BCA protein quantification of the filter-sterilized sample. These VLPs were demonstrated to be safe and immunogenic in horses. No lesions were observed at the site of injection and no side effects were identified as a result of administering either the VLP or Live attenuated vaccine (LAV). The immune response induced by AHSV 6 VLP vaccine was variable (Table 1). The neutralizing antibody titres were low and could not be detected on some days. A similar response was observed with monovalent live attenuated AHSV 6 vaccine. Following processing of the serum samples for the neutralization assay, some samples displayed toxicity (T) towards the cells in this assay.

**Table 1:** Virus neutralizing antibody titres of serum from adjuvanted AHSV-6 VLP and LAV (OBP) vaccinated horses^a^

	Horse ID	Day 0	Day 7	Day 14	Day 21	Day 28	Day 35	Day 42
AHSV-6 VLP vaccine	**#2**	0	1:2	1:2	1:4	1:4	1:4	1:8
	**#3**	0	0	1:32	1:16	1:8	1:16	1:32
	**#6**	0	T	T	1:4	T	1:2	1:4
AHSV-6 LAV (OBP)	**#9**	0	0	1:4	1:4	1:4	1:4	1:2
	**#10**	0	1:2	1:4	1:8	1:4	1:16	1:8
	**#11**	0	1:8	1:8	1:4	1:8	1:4	1:4
Bicine buffer (negative control)	**#14**	0	1:2	0	1:2	0	0	0

^a^ Sera were assayed for neutralization activity against AHSV-6. The adjuvant was Montanide™ Pet Gel A (Seppic)

T: Toxicity

## Discussion

In this study we describe the transient expression of the four major structural capsid proteins of AHSV in *Nicotiana benthamiana* dXT/FT plants and their self-assembly into homogenous and chimeric virus-like particles (VLPs). Not only were we successful in demonstrating assembly of homogenous VLPs of AHSV serotype 1, but also assembly of single, double and triple chimeric AHS VLPs, composed of a combination of capsid proteins from at least two different AHS serotypes. Transient expression of heterologous proteins in plants has a number of advantages over other expression platforms including speed, scalability, cost-effectiveness and safety [34, 38, 53]. It is thus not surprising that VLPs against a large number of diseases are currently being developed and produced in plants [35, 36]. Self-assembly of BTV VLPs in *N. benthamiana* plant cells following transient co-expression of the four BTV capsid proteins [42], prompted us to investigate whether this was also possible in the case of the closely related AHSV. Very recently the assembly of homogenous AHSV-5 VLPs was demonstrated in plants and found to elicit a neutralizing immune response in horses [44, 45]. Some promising preliminary work in our laboratory with an RNAi mutant dXT/FT *N. benthamiana*, which facilitates mammalian-like glycosylation [46], and may result in AHSV VLPs more closely resembling AHSV virions with authentic post translational modifications, resulted in the use of this dXT/FT as the expression host in this study.

We firstly wanted to determine whether we could demonstrate assembly of homogenous AHSV-1 VLPs in *N. benthamiana* dXT/FT plants. To this end the four AHSV-1 capsid proteins, VP2, VP5, VP3 and VP7, were co-expressed in the plant cells and we were able to visualise homogenous AHSV-1 VLPs, approximately 70 nm in size [1, 2]. AHSV-1 core-like particles (CLPs), approximately 60 nm in size and with a knob-like surface appearance were also visualised. The diameters of plant-expressed BTV VLPs have been reported to range from 72 to 86.8 nm whilst the BTV CLPs range from 60 to 69 nm in diameter [42, 43].

As AHS has 9 serotypes, it would be necessary to assemble VLPs of all nine serotypes and combine them into a multivalent vaccine in order to protect horses against the disease. With this in mind, we synthesized and transiently co-expressed plant-codon optimised sequences encoding the four capsid proteins of AHS serotype 7 in *N. benthamiana* dXT/FT plant cells. AHSV serotype 7 is one of the serotypes responsible for the majority of the AHS outbreaks between 1997 and 2006 [54]. Interestingly, we found that the serotype 7 capsid proteins, when co-expressed in plants, failed to assemble into AHSV-7 VLPs. This is not entirely unexpected as it has previously been shown that while the plant-expressed BTV-8 capsid proteins assemble into VLPs, the capsid proteins of the BTV-10 serotype fail to assemble [55].

We promptly embarked on a different strategy to generate AHSV-7 VLPs. As *Orbivirus*es are capable of genome segment reassortment, it is possible to exchange specific gene segments with those of another serotype and, through reverse genetics, generate ‘chimeric‘replication-deficient BTV and AHSV [49–52, 56]. It has also been shown that insect-cell expressed VP2 and VP5 outer capsid proteins of one BTV serotype assembled into chimeric BTV VLPs with the VP3 and VP7 core proteins of another BTV serotype [47, 48]. Here we aimed to generate plant-expressed, chimeric VLP-based AHS vaccine candidates by exchanging the outer capsid proteins AHSV VP2 and/or VP5 with those of a different serotype whilst retaining the conserved AHSV inner core (VP7 and VP3 proteins) of the original serotype. This strategy of generating ‘chimeric’ AHSV VLPs would reduce the number of constructs required and significantly simplify the process of generating a multivalent AHSV vaccine, composed of all nine AHSV serotypes.

We exchanged the AHSV-1 VP2 outer capsid protein, the main neutralizing antigen, with that of AHSV-7 VP2 protein, whilst retaining the VP3, VP7 and VP5 proteins of AHSV-1. We found that these capsid proteins assembled into single chimeric AHSV-1/AHSV-7 VLPs in *N. benthamiana* dXT/FT plants. In an attempt to increase particle stability of the single chimeric AHSV-1/AHSV-7 VLPs, we also exchanged the AHSV-1 VP5 protein with that of the AHSV-7 serotype. Studies have shown that the VP5 outer capsid protein enhances the conformation of VP2, thus increasing this protein’s interaction with core-like particles [57–59]. More recently, studies [51, 52] have shown that the exchange of only the VP2 segment can significantly lower the viral titre of reassortant viruses. We subsequently co-expressed the VP2 and VP5 proteins of AHSV-7 with the AHSV-1 VP3 and VP7 proteins in *N. benthamiana* dXT/FT cells. We found that the quantity of VP2 and VP5 outer capsid proteins on the double AHSV-1/AHSV-7 chimeric VLPs was greater than their quantity on the single chimeric VLPs, as deduced by the intensity of the relevant protein bands, which indicated greater stability of the double chimeric VLP. Increased stability and replicative capability of some of the Entry competent replication abortive (ECRA) AHSV serotypes was also observed following the exchange of both the VP2 and VP5 proteins, but not in all serotypes [52]. Our research also indicated that the exchange of both the VP2 and VP5 proteins originating from the same serotype does not always allow for the formation of stable VLPs. Empirical studies of the different combinations of outer capsid proteins originating from the 9 AHSV serotypes were required to determine which yielded stable, serotype-specific VLPs. The use of the double recombinant pEAQ-express vector, with the combined AHSV-1 VP7 and VP3 expression cassettes, greatly aided in facilitating these stability studies.

We report here on one of these combinations that resulted in the assembly of stable triple chimeric AHSV-6/AHSV-3/AHSV-1 VLPs in *N. benthamiana* dXT/FT plants. In order to generate sufficient quantities of VLPs for animal trials, a large scale VLP purification procedure was optimised, which included depth filtration, tangential flow filtration (TFF), density gradient centrifugation and dialysis [60]. A target animal trial was conducted to determine whether these AHSV-6/AHSV-3/AHSV-1 VLPs were able to induce a serotype-specific neutralizing antibody response in horses. The plant-produced, purified VLP inoculum was mixed with Montanide™ Pet Gel A (Seppic), identified as being a suitable adjuvant for horses [61]. Seven 6-month old foals were tested for the presence of AHSV VP7-specific antibodies via ELISA and were found to be AHSV naïve, not harbouring AHSV virus from either vaccination or infection. The ELISA test, a VP7-based assay, is capable of detecting the presence of all nine AHSV serotypes. We thus employed the serogroup-specific ELISA test instead of the serotype-specific serum neutralization test (SNT) to ensure no previous exposure of the foals to any of the AHS serotypes.

Primary and booster vaccinations were administered 28 days apart. The AHSV-6 monovalent live attenuated vaccine (LAV) served as the positive control in three horses and while bicine buffer served as the negative control in one horse. No side effects or disease clinical signs were observed following inoculation of three horses with either the VLP-based or LAV vaccine. In addition, no lesions were observed at the site of injection suggesting the plant produced, VLP-based vaccine is safe for use in horses.

Weak neutralizing antibody titres against AHSV-6 were observed in horses inoculated with both the VLP and LAV vaccines. On some days antibodies were not even detected. Variability in immune responses of the individual foals to vaccination with both these vaccines was also observed. These results are not unexpected as previous studies have shown that young foals vaccinated with the commercial polyvalent, as well as monovalent AHSV vaccines, have weak or non-detectable and variable antibody responses to all AHSV serotypes, except serotype 1, and some only seroconvert after revaccination [62]. Current OBP AHS LAV protocol recommends 2 to 3 vaccinations to obtain sufficient titres of antibodies to confer protection against all serotypes in the vaccine and a recent study [63] suggests up to eight vaccinations before full protection can be conferred. It is likely that, as with the live attenuated polyvalent vaccine, multiple vaccinations with the AHSV VLPs will be required to induce titres of neutralizing antibodies sufficient for protection. Low inconsistent reactions obtained with the negative control in this study was attributed to the presence of passively acquired, maternal neutralizing antibody in the six-month old foal. The duration of maternal antibodies against AHSV-6 has previously been found to be 115 days (3.8 months) [64], however, the small sample size (*n* = 6) may result in this period not being indicative of the actual duration. As seroconversion is considered positive when the neutralizing antibodies are at titres of 4 or higher, and no VP7-specific antibodies were detected with ELISA, negative control foal #14 is considered seronegative. In future studies we would like to increase the sample size as well as use AHSV-naïve horses that are older than 12 months in order to reduce the effect of maternal antibodies on the results.

This is the first report of plant-produced, chimeric AHSV VLPs vaccines eliciting a neutralizing immune response in horses. Chimeric VLPs are defined as having their component structural proteins originating from at least two different serotypes. Here we have shown that, not only do chimeric VLPs assemble successfully in *N. benthamiana* dXT/FT plants, but they are also able to elicit a serotype-specific immune response in the target animal. This is the first step in the development of a plant-produced, multivalent AHS vaccine, formulated with a combination of homogenous and chimeric VLPs, and capable of inducing species-specific immunity against all nine AHS serotypes.

## Conclusions

In the present study we report on the development of a novel virus-like particle (VLP)-based vaccine, produced in plants, to be used as a preventative measure against African horse sickness disease in horses. We demonstrate that chimeric VLPs, composed of proteins from different AHSV serotypes and produced in plants, are capable of inducing serotype-specific neutralizing antibodies when injected into horses. This new generation vaccine may be utilized as a safe alternative to the current live attenuated vaccine in a control strategy against this devastating, OIE notifiable disease.

## Methods

### Constructs

Gene sequences, encoding the VP2, VP5, VP3 and VP7 proteins of AHSV serotype 1 (Genbank accession numbers AM883165, FJ183369, FJ183366, AM883171, respectively), the VP2 (Genbank accession number AY163330) and VP5 (Genbank accession number JQ742011) proteins of AHSV serotype 7, the VP5 protein of AHSV serotype 3 (Genbank accession number DQ868777 and the VP2 protein of AHSV serotype 6 (Genbank accession number DQ868774.1) were codon optimised for optimal expression in *N. benthamiana* plant cells and synthesized with AgeI and XhoI sites at the 5′ and 3′ termini, respectively (BioBasic Inc., Canada).

The VP2, VP5, VP3 and VP7-encoding nucleotide sequences were subsequently cloned into the pEAQ transient expression vectors [40], made available to the CSIR under a licence agreement from Plant Bioscience Limited (PBL), UK. More specifically, sequences encoding the AHSV-1 VP2, VP5, VP3 and VP7 proteins were firstly cloned into the intermediate pEAQ vectors FSC5 or FSC6 via directional AgeI/XhoI restriction enzyme-based cloning. The AHSV-1 VP5- and AHSV-1 VP7-encoding expression cassettes were subsequently individually cloned into the pEAQ express vector via directional AscI/SbfI restriction enzyme-based cloning. The AHSV-1 VP2 and VP3-encoding genes were inserted into the pEAQ-HT vector individually via directional AgeI/XhoI restriction enzyme-based cloning. The AHSV-7 VP2 and AHSV-7 VP5 encoding sequences were cloned individually into the pEAQ-HT vector via directional AgeI/XhoI restriction enzyme-based cloning. Cloning of the sequences encoding the AHSV-3 VP5 and AHSV-6 VP2 proteins individually into the pEAQ-HT vector was performed using the In-Fusion HD® cloning kit (Clontech), according to the manufacturer’s instructions.

To generate the dual recombinant plasmid pEAQ-express-AHSV-1VP3-AHSV-1VP7, the AHSV-1 VP7 encoding sequence was firstly cloned from the pEAQ-express-AHSV-1VP7 plasmid into the pEAQ-HT vector via directional AgeI/XhoI restriction enzyme-based cloning. The VP7-encoding expression cassette was subsequently excised from pEAQ-HT-AHSV-1VP7 using the AscI/PacI enzymes and cloned into the compatible MluI/AsiSI sites of the pEAQ-express vector. The VP3-encoding expression cassette was transferred from the pEAQ-HT-AHSV-1VP3 plasmid into the newly generated pEAQ-express-AHSV-1VP7 plasmid via AscI/PacI mediated restriction enzyme based cloning. Recombinant plasmids were transformed into electrocompetent *Escherichia coli* DH10B bacterial cells and their sequences verified via dideoxy Sanger DNA sequencing (Inqaba Biotechnical Industries (Pty) Ltd).

### *Agrobacterium*-mediated infiltration of *Nicotiana benthamiana* plants

All expression constructs were transformed into *Agrobacterium tumefaciens* LBA4404 bacterial cells (Invitrogen) by electroporation and propagated at 28 °C in YMB media (0.1% yeast extract, 1% Mannitol, 1.7 mM NaCl, 0.8 mM MgSO_4_.H_2_0, 2.2 mM K_2_HPO_4_) containing 50 μg/ml Kanamycin, 50 μg/ml Rifampicin and 50 μg/ml Streptomycin. Cryopreserved LBA4404 cells, containing the pEAQ-HT vector or the pEAQ-HT-GFP recombinant plasmid (G. Lommonosoff, John Inness Centre, UK), served as negative and positive controls, respectively. Seeds of the *N. benthamiana* dXT/FT plants were originally acquired from Icon Genetics GmbH under the auspices of a Material transfer agreement. Transient expression of the AHSV capsid proteins in *N. benthamiana* dXT/FT plants was accomplished by *Agrobacterium*-mediated infiltration of their leaves.

LBA4404 agrobacterial cultures grown overnight were harvested via centrifugation (7000×g for 7 min at 10 °C) and pellets resuspended in freshly prepared MMA infiltration buffer (10 mM MES hydrate; pH 5.6, 10 mM MgCl_2_, 100 μM 3, 5-Dimethoxy-4-hydroxy-acetophenone). The bacterial suspensions were combined in 1:1:1 ratios for VLP assembly while the pEAQ-express-AHSV-1VP7-AHSV-1VP3 suspension was sufficient for CLP assembly. The combinations were subsequently diluted with MMA buffer such that the final OD_600_ = 0.45–0.5 or, in the case of plants being infiltrated for animal trials, an OD_600_ = 2. The leaves of four-week-old *N. benthamiana* dXT/FT plants were syringe-infiltrated with the bacterial combinations or the pEAQ-HT/pEAQ-HT-gfp bacterial suspensions. The plants were incubated at 27 °C.

### Protein extraction and small scale VLP purification

Agroinfiltrated *N. benthamiana* leaves were harvested 8 days post-infiltration (d.p.i). The leaf tissue was extracted immediately in 3 volumes of VLP extraction buffer (50 mM Bicine, pH = 8.4, 20 mM sodium chloride (NaCl), 0.1% (w/v) Sodium lauroyl sarcosine (NLS), 1 mM Dithiothreitol (DTT) (ThermoScientific), 0.2% Protease inhibitor cocktail P2714 (Sigma Life Science)) in a multipurpose juice extractor (MATSONE). The DTT and Protease inhibitor cocktail were freshly prepared according to the manufacturer’s instructions and added to the extraction buffer just prior to use. The plant extract was clarified via centrifugation (8000×g; 10 min; 4 °C).

AHS virus-like particles (VLPs) or core-like particles (CLPs) were purified using density gradient centrifugation using either sucrose or iodixanol density media. Sucrose gradient solutions (60–30%) were prepared by dissolving ultra-high quality sucrose (Sigma Life Science) in VLP buffer (50 mM Bicine, pH = 8.4, 20 mM NaCl) and layered into gradients of 1 ml 10% incremental steps. Iodixanol solutions (60–20%) were prepared by diluting 60% iodixanol (OptiPrep™ Density Gradient Medium) (Sigma-Aldrich) in VLP dilution buffer and layered into gradients of 10% incremental steps. The clarified cell lysates were layered on top of the sucrose gradients and centrifuged in a SW-41Ti rotor (Beckman Coulter) at 32,000×g for 2 h; 10 °C. The 55–35% sucrose layers, or 35–25% iodixanol layers, were harvested in 500 μl fractions via a Minipuls2 peristaltic pump (Gilson). Ten microlitres of each fraction was subsequently analysed for protein content by denaturing 10% SDS-PAGE (BioRad TGX Stain Free™ Fast Cast™), according to the manufacturer’s instructions. The Precision Plus Protein™ Western C™ standard (Bio-Rad) or the PageRuler Plus Prestained protein ladder (Thermo Scientific) were used as size markers. The gels were either subjected to 0.1% Coomassie Brilliant Blue G250 staining (Merck) and subsequent destaining, or to immunoblotting procedures with a guinea pig anti-AHSV-7 polyclonal serum (kindly donated by Onderstepoort Biological Products (OBP)). Protein samples were immunoblotted onto a PVDF membrane within the Trans-blot® Turbo™ Transfer Pack (Bio-Rad) using the Trans-Blot®Turbo™ Transfer system (Bio-Rad) mixed MW application, according to the manufacturer’s instructions. The membranes were blocked and incubated with the polyclonal antiserum (1:300 dilution in 3% Bovine serum albumin (Roche)) overnight at 4 °C. Following washing Rabbit anti-Guinea Pig IgG H&L (HRP) conjugate (Abcam ab6771) (1:5000 dilution) and Precision Protein™ StrepTactin-HRP Conjugate (Bio-Rad) (1:10000 dilution) secondary antibodies were incubated with the membrane for 1 h at room temperature. Clarity Western ECL chemiluminescent substrate (Bio-Rad), according to the manufacturer’s instruction, was added to detect the proteins and imaging was performed using the Chemi Hi Resolution application in the ChemiDoc™ MP Imager (Bio-Rad).

### Protein confirmation using LC-MS/MS-based peptide sequencing

Following electrophoresis of density gradient fraction samples on precast denaturing 4–12% Bolt™ Bis-Tris Plus polyacrylamide Gels (Thermo Fischer Scientific), according to the manufacturer’s instructions, and Coomassie Brilliant Blue G250 staining and destaining, candidate protein bands of approximately the correct size were excised from the gel and sent for LCMS-MS peptide sequencing analysis, previously described by our colleagues [65]. Briefly, the protein bands were in-gel trypsin digested, resuspended in 2% acetonitrile/0.2% formic acid and analysed using a Dionex Ultimate 3000 RSLC system coupled to an AB Sciex 6600 Triple TOF mass spectrometer. The obtained MS/MS spectra were compared with the Uniprot Swissprot protein database by using Protein pilot v5, which makes use of the Paragon search engine (AB Sciex). Proteins with a threshold above ≥99.9% confidence were reported.

### Transmission electron microscopy (TEM)

VLPs from the 55–45% sucrose fractions were visualised by adsorbing samples onto carbon-coated holey copper grids (5 min) and subsequently stained with 2% sodium phosphotungstate, pH = 7 for 30 s. The grids were air dried and subsequently imaged in a JEM-2100 Transmission electron microscope (JEOL). VLPs from 35 to 25% iodixanol fractions were visualised by adsorbing samples onto carbon-coated holey copper grids (5 min) and stained with 3% uranyl acetate, pH = 4.2 for 20 s. Grids were air dried and imaged in a CM-10 Transmission electron microscope (Philips). The diameters of the particles visualised on the grid were measured using the measure tool on the Gatan Digital Micrograph software. Thirty five particles of each type were measured and the mean diameter calculated.

### Large scale AHSV VLP purification for animal trials

Leaves of *N. benthamiana* dXT/FT plants were infiltrated with the appropriate recombinant *Agrobacteria* combinations, as detailed previously (7.2), and plants incubated at 27 °C. Infiltrated leaves were harvested 8 d.p.i, weighed and processed through a juice extractor (MATSONE) with 2 volumes of VLP buffer (20 mM NaCl, 50 mM Bicine, pH = 8.4). Protease Inhibitor cocktail P2714 (Sigma Life Science) was added to the buffer immediately prior to use, according to the manufacturer’s instructions. The cell extract clarified via centrifugation (8000×g; 10 min; 4 °C) and filtered through a Sartoclean GF sterile midicap (3 + 0.8 μM) depth filter (Sartorius Stedim Biotech GmbH) and subsequently a 300 K Minimate™ Tangential Flow Filtration (TFF) Capsule (Pall Life Sciences) until the extract was reduced to one tenth of its original volume. Following centrifugation through 60–20% Iodixanol density gradients (32,000×g; 2 h; 10 °C), the 35–25% iodixanol fractions were combined and dialysed against sterile buffer (20 mM NaCl, 50 mM Bicine, pH = 8.4) overnight in SnakeSkin™ dialysis tubing (Thermo Fischer Scientific). The sample was filter-sterilized through a 0.45 μM + 0.2 μM Sartobran 300 Sterile capsule (Sartorius Stedim Biotech GmbH) and 5 % Montanide™ Pet Gel A (Seppic) was added as an adjuvant. Samples were stored at 4 °C prior to use in the animal trial. The protein content of the filter-sterilized sample was quantified by using the Micro BCA™ Protein Assay kit (Thermo Fisher Scientific), according to the manufacturer’s instructions, Aliquots, taken throughout the course of the purification procedure, were analysed for protein content by iodixanol centrifugation and fraction analysis via denaturing SDS-PAGE. VLPs were visualised via TEM. The aliquot of the concentrated filter-sterilized sample was diluted 1 in 10 prior to centrifugation and TEM.

### Animal care and animal facility

Healthy horses (*Equus caballus) of both sexes*, between 6 and 12 months, were sourced from a farmer, tested for AHSV status via Enzyme-linked immunosorbent assay (ELISA) according to the protocol described in the Office International des Epizooties (OIE) Manual of Diagnostic Tests and Vaccines for Terrestrial Animals [66] and treated against any other ailments prior to the start of the trial. The animals were housed in closed stable 166 at OBP for the duration of the trial. Large animals were handled *and cared for according to standard operating procedures outlined by the Experimental Animal Unit. Briefly, h*orses were fed Epol Riders pellets. Eragrostis and clean water ad lib. The horses were not euthanized and returned to the farmer upon completion of the animal trial on day 42.

### Immunogenicity trial in horses

Seven AHS-naïve foals were randomly allocated into one test, one positive control and one negative control group for the immunogenicity trial, stabled in closed stable 166 at OBP and handled according to regulatory guidelines and standard operating procedures outlined by the Experimental Unit. The welfare of the horses was monitored daily by competent staff according to a standard welfare monitoring sheet, with instructions to report any adverse effects immediately to the project leader. The inoculation site (subcutaneous route) was monitored for recordings of local reactions (onset and duration) and body temperatures recorded for a minimum of 3 days before vaccination and for 14 days post vaccination. Vaccination and bleeding of animals was according to standard operating protocols by trained and competent staff and done in the morning in the closed stables. Bleeding via venepuncture was performed with 19 gauge needles in 10 ml yellow vacutubes (Labchem Sdn Bhd) and sent the same day for laboratory analysis. The presence of AHSV VP7-specific antibodies in the foals was assayed by an Indirect Enzyme-linked immunosorbent assay (ELISA), performed by ARC-OVR according to the protocol described in the Office International des Epizooties (OIE) Manual of Diagnostic Tests and Vaccines for Terrestrial Animals [66]. The statistical requirement for the minimum number of animals per group was 8 and this number is also required for vaccine data registration. However, previous experimental research using a reduced number of animals has shown 3 animals are sufficient to achieve the required scientific objectives [68, 69].

In order to obtain preliminary data for this proof of concept study, three foals were injected subcutaneously into the inner thigh with sterile Montanide™ Pet Gel A adjuvanted AHSV-1/AHSV-3/AHSV-6 VLPs (final volume of 2 ml containing 220 μg of total protein) on Day 0. Two hundred microgram total protein has previously been used to vaccinate horses against homogenous AHSV-5 VLPs [45]. One foal was inoculated subcutaneously into the inner thigh with sterile Montanide™ Pet Gel A adjuvanted bicine buffer as a negative control. Three horses were inoculated with 1x10E4 per dose monovalent AHSV-6 live attenuated vaccine subcutaneously into the inner thigh as a positive control. Subcutaneous administration of the live attenuated AHSV vaccine is routine and this same route was used for the administration of the VLP-based antigen. The animals were boosted using the same route of administration and same antigen dose on day 28 of the immunization schedule and serum samples taken on days 0, 7, 14, 21, 28, 35 and 42. The presence of neutralizing antibodies in each experimental animal was determined by performing a virus neutralization assay on homologous AHSV-6 monovalent viral strain [67]. This assay was performed by OBP according to the protocol described in the OIE Manual of Diagnostic Tests and Vaccines for Terrestrial Animals [66]. The 50% end-point titre of the serum is calculated by the Spearman–Kärber method and expressed as the negative log_10_.

## Data Availability

The datasets used and/or analysed during the current study are available from the corresponding author on reasonable request.

## Abbreviations

AHS: African horse sickness
AHSV: African horse sickness virus
ARC-OVR: Agricultural Research council-Onderstepoort Veterinary Research
BT: Bluetongue
BTV: Bluetongue virus
CLP: Core-like particle
CSIR: Council for Scientific and Industrial Research
d.p.i: days post infiltration
DAFF: Department of agriculture, forestry and fisheries
DIVA: Differentiation between infected and vaccinated animals
DTT: Dithiothreitol
*E.coli*: *Escherichia coli*
ELISA: Enzyme-linked immunosorbent assay
gfp: green fluorescent protein
HRP: Horse radish peroxidase
LAV: Live attenuated virus
LC-MS/MS: Liquid chromatography-mass spectrometry
OBP: Onderstepoort Biological Products
OIE: World organisation for animal health
PBL: Plant Bioscience Limited
SDS-PAGE: Sodium dodecyl sulphate polyacrylamide gel electrophoresis
TEM: Transmission electron microscope
TFF: Tangential flow filtration
UK; RNAi: RNA interference
VLP: Virus-like particle
VP3/7/5/2: Viral protein 3/7/5/2
